# Sectional Medicine

**Published:** 1849-01

**Authors:** 


### SECTIONAL MEDICINE.

We have never been of the number who contend, that a physician ought to be educated in the region of country where he is to practice his profession, and what has been urged of late years in support of the doctrine has only served to confirm our convictions of its unsoundness. The faculty of Hampden-Sydney College have been amongst its most strenuous advocates. Recently, Dr. Cullen, the professor of Theory and Practice, found it necessary to relinquish the duties of his chair on account of indisposition, and Dr. Clymer, of Philadelphia, has been invited to take his place for the present session. The appointment is a most judicious one; but what becomes of the theory, that students to treat southern diseases must study in a southern school, when their instructor in the practice of medicine is a northern man?

